# Correlation between missed abortion and insertional translocation involving chromosomes 1 and 7

**Published:** 2012-01

**Authors:** Neveen Ashaat, Ahmed Husseiny

**Affiliations:** 1 Department of Zoology, Faculty of Women for Arts, Science and Education, Ain Shams University, Cairo, Egypt.; 2 Department of Obstetrics and Gynecology, Maternity Hospital, Ain Shams University, Cairo, Egypt.

**Keywords:** *Missed abortion*, *Maternal chromosomal aberrations*, *Fluorescence In Situ Hybridization*

## Abstract

**Background**: Missed abortion (Silent miscarriage) is defined as intrauterine fetal death before twenty weeks gestation. One of the most common causes of early missed abortions (before 10 weeks gestation) is cytogenetic abnormalities.

**Objective**: To asses if there is a correlation between chromosomal aberrations (especially in chromosome 7) and missed abortion among at least two generations.

**Materials and Methods**: After exclusion of direct causes of missed abortion, this study included 60 women (the study group) who had first trimestric missed abortion and 30 healthy women who did not suffer from any diseases during their pregnancy and had apparently normal outcome (the control group). All cases were diagnosed; the blood and tissue samples were collected from the mothers and abortuses from the Department of Obstetrics and Gynecology, Maternity Hospital, Ain Shams University. Cytogenetic analyses were performed by using conventional technique and G/T banding techniques and Fluorescence In Situ Hybridization (FISH) analysis with a whole chromosome 7 painting probe (WCP7) and a 7q subterminal probe (7q36, qter), prepared by chromosome micro dissection technique was used for confirming the specific chromosomal abnormality.

**Results: **<insertional translocation between chromosomes 1 and 7 (46, XX, ins. (1; 7) (p32; q32.35). This insertion has appeared in two families and among two generations, and in one family among three generations.

**Conclusion: **Chromosome 7 insertional translocation is a possible autosomal dominant inherited trait and may cause missed abortion.

## Introduction

Missed abortion (miscarriage) can be divided into embryonic (preclinical) or fetal abortion. Embryonic miscarriage is defined as an embryo with crown rump length of more than or equal to 5cm without cardiac activity, fetal miscarriage is defined as a fetus of 7-20 weeks size with negative cardiac activity (1). There is a big volume of literature describing that cytogenetic abnormality is detected in 31% of early missed abortion (2, 3). 

Genetic factors including chromosomal disorders, single gene defects, and multifactorial factors account for 3.5-5% of the causes of recurrent missed miscarriage. In about 4% of couples with recurrent miscarriage, one partner carries either a balanced reciprocal translocation, in which there is an exchange of two terminal segments from different chromosomes, or a robertsonian translocation, in which there is centric fusion of two acrocenteric chromosomes (4).

Carriers of a balanced reciprocal translocation are phenotypically normal, but 50-70% of their gametes and hence embryos are unbalanced because of abnormal segregation at meiosis. The reproductive risk conferred by chromosome rearrangements is dependent on the type of rearrangement and whether it is carried by the woman or her male partner (5-7). Thus, cytogenetic studies are of interest in the first trimester missed abortions, particularly among couples with recurrent miscarriage. 

The presence of chromosomal abnormality in miscarriages may explain the reason for the pregnancy loss. The analysis of aneuploidies, translocations and other gross structural aberrations of the chromosomes have greatly helped to determine the etiology in the majority of cases of missed abortion (8, 9).

Cytogenetic studies have revealed that fetal chromosome abnormalities account for about 50% of first trimester and near 30% of second trimester pregnancy losses. Most of these abnormalities are numerical chromosomal aberrations (86%) and a low percentage is structural chromosomal aberrations (6%) or others, including chromosome mosaicism (8%) (9, 10). 

In the present article we have tried to evaluate the relationship between abortal and maternal chromosomal abnormalities especially the genuine observation of chromosome 7 abnormality as a possible cause of missed abortions.

## Materials and methods

The present study is retrospective cohort study including Ninety women who visited the department of obstetrics and gynecology at Ain Shams University Hospital, Cairo, Egypt, a tertiary referral center serving 1500 births/year. These cases were classified into two main groups, the first including 30 healthy women who delivered healthy babies and don’t have any known genotypic or phenotypic abnormality regarding the parents nor their families, this group was represented as the control group (CG). 

The second group including 60 women were diagnosed as cases of first trimesteric missed abortion, the inclusion criteria were any woman having first trimesteric missed abortion and the exclusion criteria were any patient with any possible cause of this missed abortion as uterine cavitary lesion, abnormal hormonal profile especially exclusion of (DM, luteal phase defect, thyroid dysfunction), laboratory proven antibodies to cardiolipin and phosphatidyl serine and lupus anticoagulant or recent infection were excluded, this group was represented as the studied group (SG). 

Because of the high failure rate of post-abortal and post-stillbirth karyotyping, the Working Party of the Royal College of Pathologists (2010) recommends that multiple samples be collected, usually placenta and full thickness skin. Consideration should also be given to collecting a specimen in utero before the termination process begins. 

Because we were dealing with early abortions, products of conception were obtained from the studied group (60 women) who underwent evacuation by curettage or suction at Ain Shams University Maternity Hospital. The abortal tissue was immediately collected on a special nutrient media [RPMI] in a sterile container, this media is enriched with antibiotics and antimycotics. 

In cases of early abortion <7 weeks pregnancy meticulous choice of the abortal tissue was done to avoid maternal tissue karyotyping (Decidua). In advanced pregnancies direct fetal tissue was selected (cord-skin) (8). 

Cytogenetic analyses were performed at Cytogenetic laboratory, Department of Zoology, University of Women College for Art, Science and Education, Ain Shams University, Cairo, Egypt. Fluorescence in situ hybridization (FISH) with a whole chromosome 7 painting probe (WCP7) and a 7q subterminal probe (7q36، qter), was performed at Medical Lab., between April 2008 to December 2009. 

For each case the following were conducted:

- Three generations pedigree construction and analysis including consanguinity, similar conditions and other affected members in the family.
- Complete history taking that includes parental occupation, obstetric history, exposure to drug intake, fever, recent infection and trauma.
- Physical examination, pelvic ultrasound, laboratory workup (thyroid-stimulating hormone, anti-cardiolipin antibodies IgM and IgG and lupus anti-coagulant and one hour postprandial glucose level) to exclude other causes of missed abortion. All couples had normal phenotypes, mentality and external genitalia.
- The data collection was focused on the maternal characteristics at the time of chromosome analysis.

Cytogenetic analyses were performed by using conventional technique and G/T banding techniques were performed to all parents (blood culture) and their abortuses (tissue culture), as well as, chromosomal analysis of one of the female relatives of the mother (grandmother or aunt) was done (11, 12). Karyotypes were recorded according to the recommendations of the International Standing Committee on Human Cytogenetic Nomenclature (13). 

Fluorescence In Situ Hybridization (FISH) analysis with a whole chromosome 7 painting probe (WCP7) and a 7q subterminal probe (7q36، qter), prepared by chromosome microdissection technique was used for confirming of the specific chromosomal abnormality. The slides for FISH were stored at -20^o^C.

**Preparation of probes**

The probe of WCP7 was generated by chromosome microdissection. The procedure was performed essentially as Guan *et al* method (9). 7q subterminal probe (7q36, ter) was presented by Guan (9). The specificity of these probes was determined by FISH with normal metaphase.

**Fluorescence in situ hybridization**

FISH on the metaphases of the cases and normal individual were performed as described by Guan (9). Briefly, the probes were labeled with biotin-16-dUTP (Abbott, Illinois, USA). For each hybridization, about 100mg of probe was used in 10ml hybridization mixture (containing 55% formamide, 2xSSC, and 10% dextran sulfate), which was denatured at 75°C for 5min. 

The slide with metaphase spreads was denatured in denaturing solution (70% formamide and 2xSSC) at 70°C for 2min, and then hybridized with probes in a moist chamber overnight. The slides were then washed two times in 50%formamide, 2xSSC, two times in 0.1xSSC, and one time in 4xSSC. All washes were performed at 45°C. 

The hybridization signal of the biotin-labeled probe was detected by avidin-FITC and amplified with anti-avidin conjugate (developed in mouse) and anti-mouse IgG conjugated with FITC (Sigma, USA). Propidium iodide (PI, 0.5mg) or 4, 6-diamidino-2-phenylindole (DAPI, 0.1mg/ul) in an anti-fade solution was used as a counterstain. 

***Statistical analysis***

Differences between couples and abortuses were statistically analyzed using the χ^2^ test for categorical variables. P-values <0.05 were considered significant. All statistical analyses were performed using SPSS version 12. 

**Financial support**

This study is self funded by the authors. 

## Results

In the present study, we classified the cases that were ninety women, into two main groups, the first one included 30 healthy pregnant women who delivered healthy babies, their mean age was (26.5±3.2) years, and this group was represented as the control group (CG). 

The second group including 60 women who were diagnosed as cases of first trimesteric missed abortion, and their mean age was (30.6±3.6) years. The mean gestational age of the miscarriages was (8.3 weeks), 12 out of them (20%) suffered from the recurrent abortions with three or more consecutive miscarriages, and this group is represented as studied group (SG). 

G-banded chromosomes and family pedigree were analyzed from the three generations of our CG and SG. Cytogenetic analysis revealed that chromosomal abnormalities in the control group (CG) were 3.33% and 2% of structural chromosomal abnormalities were detected in 1^st^ generation and 2^nd^ generation, respectively, while, all their delivered babies had normal karyotypes. In addition, there was neither any dominantly inherited trait nor disorder through many generations in each family of CG. 

In our studied group, chromosomal analysis was carried out through three generations included the probands or abortuses, after the G-banding analyses, we found forty out of sixty (66.7%) of the studied abortuses had normal karyotype and the remaining twenty abortuses (33.3%) had abnormal karyotype, sixteen out of twenty (26.6%) abnormal abortuses' karyotypes had numerical aberrations and the rest four (6.7%) had structural aberrations, (Table I). 

Also we found highly statistical significance between maternal and abortal karyotype (p0.005), where abnormal maternal karyotype was detected among twelve cases (20%) out of 60 who had normal paternal karyotypes, five of them (8.33% of the study group) had insertional translocation between chromosomes 1 and 7 (46, XX, ins. (1;7) (p32; q32.35), three out of these five mothers suffered from recurrent missed abortion (Table II). 

**Figure 1:**
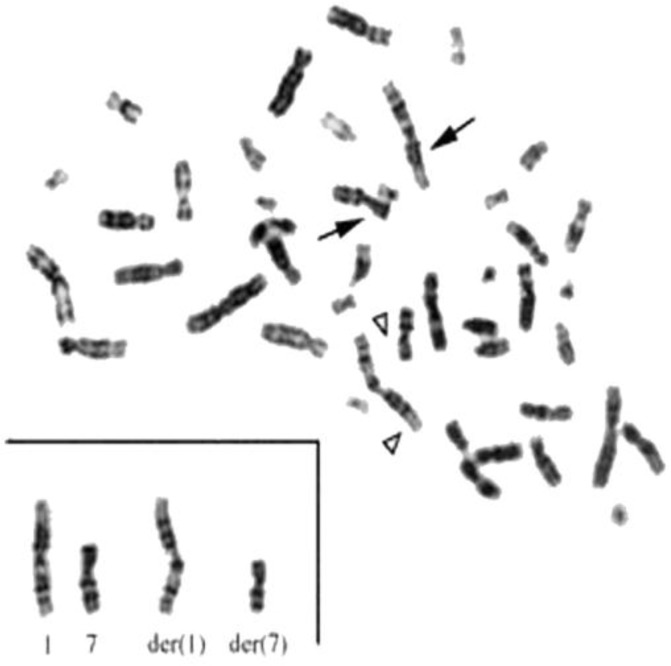
Partial karyotype of the case. A seeming terminal deletion was seen on one chromosome 7. The normal chromosomes are marked by arrows. The derivative chromosomes are marked by arrowhead

By using G-banding technique, we detected five mothers (8.33%) who had deletion in chromosome 7q with an extra band on chromosome 1, these two main findings might be a possible terminal deletion of chromosome 7q34،qter, and a possible presence of two break sites in 1p32 and 7q34, (Figure 1). 

**Figure 2:**
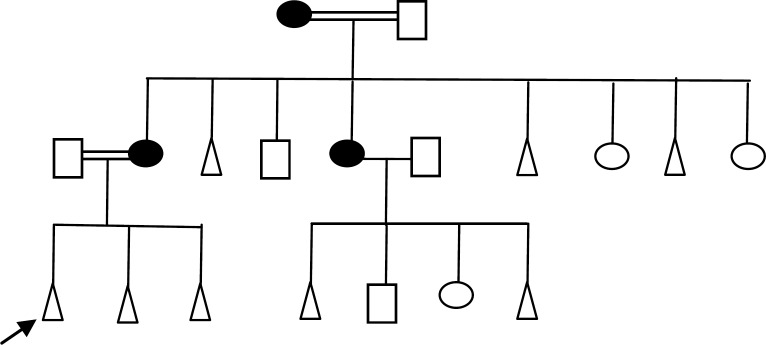
Family Pedigree showed dominantly inherited trait through many generations of one family, females are insertion carriers [46, XX, ins. (7:1) (q32.35; p32)]

Due to the absence of clinical features, the wives may be considered as balanced carriers. Where this insertion has appeared in two families among two generations (grandmother and mother of proband), also this chromosomal abnormality was detected in one family among the three generations (1.67%), through three generations that were represented by the grandmother, aunt and the mother of proband (Figure 2). Based on the G-banding technique alone, it was difficult to affirm the abnormality of chromosome 1, consequently, FISH technique is carried out to reveal this abnormality. FISH with WCP7 clearly documented homogeneous yellow hybridization signals along the normal chromosome 7 and the shorter abnormal chromosome 7 in all the metaphases of the previous cases. 

**Figure 3:**
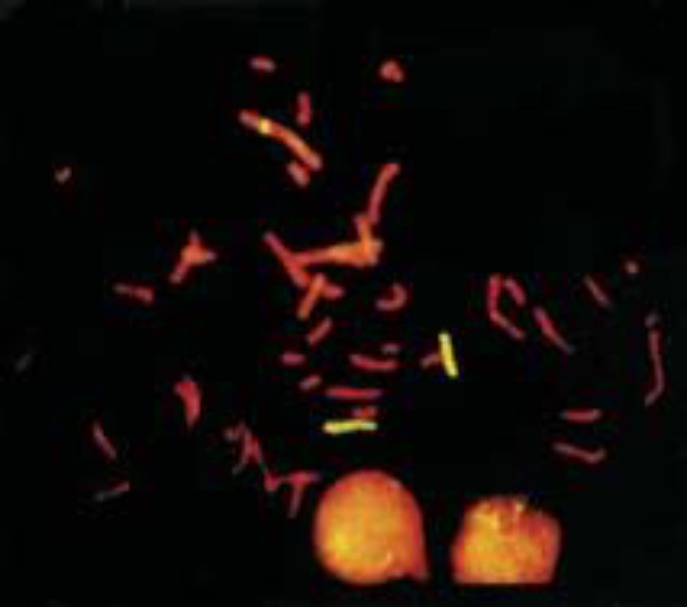
FISH examination of the case using WCP7 .There is an additional hybridization signal on derivative 1 besides two signals of chromosome 7. It showed that the case was an insertional translocation carrier. The red is counterstain color of PI, The yellow represented the hybridized signals

**Figure 4:**
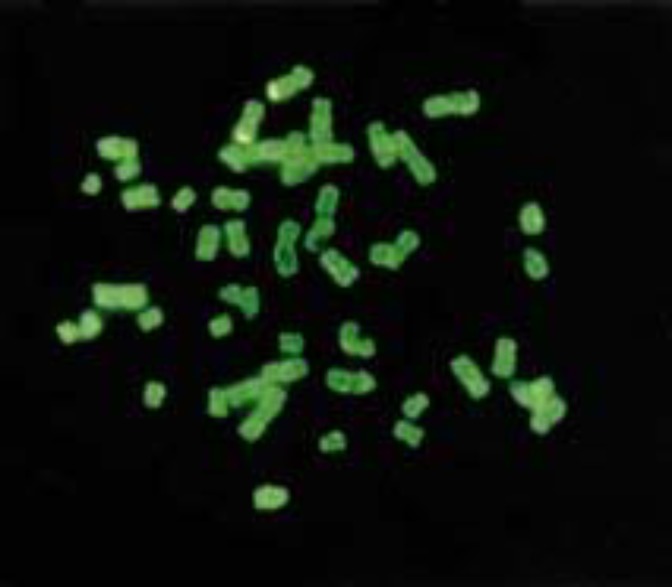
FISH examination of the case using 7q subterminal probe. There were no additional signals on other chromosomes except on chromosomes 7 .It showed the region 7q36 --qter was not inserted into the chromosome 1. The green is counterstain color of DAPI. The kelly represented the hybridized signals

Additionally, there was a positive yellow signal band within the center of chromosome 1p (Figure 3), implying that a fragment of 7q inserted into 1p32. FISH results with the 7q subterminal probe showed specific signals on both normal and abnormal chromosome 7, but no signal on chromosome1 (Figure 4). 

FISH technique showed that derivative chromosome 7 contains the region 7q36, qter and the band 7q36 was not inserted into chromosome 1p. From the results obtained by both G-banding and FISH technique, it may be concluded that 7q32, q35 was deleted from chromosome 7 and inserted into 1p32. 

In addition, the abnormal chromosome 7 was not a terminal deletion but an interstitial deletion. The insertion translocation of these cases was still a three-break rearrangement. Three break sites were 7q32 and 7q35 and 1p32. In our study, we observed a high statistical significant difference between aneuploidy in the maternal lymphocytes and those in the control group. 

The increased proportion of sporadic aneuploid cells (over 10%) was detected in 26 mothers (43.33%), (χ²=18.28, p<0.001), but there was no statistically significant difference among consanguineous marriage and family history compared to control group.

**Table I:** Abortal karyotypes in the studied group

**Karyotype**			**Frequency**	**Aneuploidy rate (%)**	**Total**	**%**
Normal karyotypes					40	66.7%
	46,XX		28	0%		
	46,XY		12	0%		
Numerical aberrations					16	26.6%
	45,X		5	100%		
	47,XX (16)		3	100%		
	47,X(10;20)		1	100%		
	48,XX(20,2)		1	100%		
	56,XXY(4;6;8;10;14;17;18;21;22)		1	100%		
	92,XXYY		1	100%		
	48,XY(16,22)		1	100%		
	69,XXY		1	100%		
	45,XX(-20)		1	100%		
	48,XX (13:20)		1	100%		
Structural aberrations					4	6.7%
	46,XX, t(7:1)(q32:q23)		2	0%		
	46,XY, dup.21		2	0%		

**Table II:** Abnormsl maternal karyotypes of the studied group (SG).

**Abnormal maternal karyotype**		**Frequency**	**Percentage**
46,XX, ins(7:1)(q32.35;p32)		5	8.33%
46,XX, del.7q33		2	3.33%
46,XX, del.8q23		2	3.33%
46,XX, del.1q25		2	3.33%
46,XX, dup.20q12		1	1.67%
Total no. of abnormal maternal karyotype		12	20%
2	6.923		
P	0.005 Highly statistically significance		

## Discussion

Approximately 15-20% of clinically recognized pregnancies are generally subjected to spontaneous abortion, mostly during first trimester (14). Most of these abortions are early missed abortions, defined as irregular pregnancy sac in which the disintegrating embryo has not developed beyond few weeks on two consecutive ultrasound examinations at an interval of one week. Several etiologies such as chromosomal abnormalities, hormonal imbalances, polycystic ovarian syndrome, immunological causes and uterine anomalies have been attributed (15). 

In 2006, Campana *et al* (16) reported in their distinguished work that couples who have balanced or unbalanced structural chromosomal abnormality as the carrier, can lead to the fetus being miscarried, a stillborn child, or a child born with major congenital defects and severe mental handicap. 

Interestingly, abortuses of carrier couples can have a normal karyotype, as in present study 66.7% of the abortuses had normal karyotypes and the remaining twenty abortuses (33,3%) had abnormal karyotype, 16 out of the twenty (26.6%) abortuses' karyotypes had numerical aberrations and the rest four (6.7%) had structural aberrations. These data concur with guidelines for the management of recurrent miscarriage which recommend chromosomal analysis in both partners as well as their abortuses (9, 12, 16). This also was in agreement with Byrne and Ward (1994) (4), who suggested that abortal chromosomal abnormalities (mostly aneuploidy) account for approximately 50% of fetal losses between 8 to 15 weeks. 

And also, in 2008, Ashaat (17) found a statistically significant correlation between the proportion of aneuploid cells which was found in the maternal lymphocytes and the proportion of aneuploid cells in the abortus, (χ²=10, p<0.01). This data support the hypothesis that parental somatic non disjunction and the proportion of aneuploid cells in the abortus are interrelated. 

On the other hand, the occurrence of chromosome insertions or insertional translocation is estimated to be less than 1/5000 in newborns. This result from three-break rearrangements, with the deleted fragment between the first two breaks inserting into the third break (10, 18-20). 

These chromosome insertions are difficult to detect by conventional cytogenetic analysis alone. Compared with a simple reciprocal translocation with only two breaks, this kind of abnormality is rarely observed. Furthermore, most insertional carriers were identified among those who had offspring with abnormal phenotypes and few were found directly in couples with a history of recurrent miscarriage (20, 21). 

In the present study, among the studied group we detected 8.3% of mothers who suffered from missed abortion, three of them (5%) suffered from recurrent missed abortion. By using G- banding technique we found all of them had a deletion in chromosome 7q, with an extra band on chromosome 1, that might be a possible terminal deletion of chromosome 7q34، qter, and the second, might be a possible two break sites in 1p32 and 7q34. 

This result was supported by several studies which revealed that there is maternal insertional translocation associated to recurrent missed abortion (18, 22-25).

In 2008, Ashaat (17) mentioned that 10% of studied women who suffered from primary recurrent abortion had cryptic translocation between chromosomes 7 and 1. She explained it as insertional translocation after magnification of normal and abnormal chromosomes (1 and 7) and drawing their idiograms, Consequently, FISH technique is carried out to reveal this abnormality in the current study, which confirmed and supported the present results. 

Additionally, with the help of FISH, some chromosome insertions have also been identified (19, 20). In some instances, only one derivative chromosome may be identified by routine G-banding, but the others cannot be visualized. Nevertheless, the results of this study are of great importance in Egypt, because we detected and confirmed by using FISH technique, the presence of inherited insertional translocation between chromosomes 7 and 1 [46, XX, ins. (7:1) (q32.35; p32)], which may be a cause for missed abortion and recurrent missed abortion. 

What was novel in this study is that we detected this abnormality in one family, through three generations; also, this abnormality appeared in two families through two generations that it is often possible to trace an autosomal dominant inherited trait. 

So, we suggest that insertional translocation between chromosomes 7 and 1 is a possible cause of missed abortion. Therefore genetic counseling and performing high quality chromosome analyses on females who have missed abortion or recurrent missed abortion are very important to detect and diagnose a main possible cause of abortion. As well as, performing chromosomal analysis for two generations at least from their maternal female relatives is also necessary for the risk assessment. 

## References

[1] Boue A, Boue J (1985). Cytogenetics of pregnancy wastage. Adv Hum Genet.

[2] Azim M, Khan AH, Khilji ZL, Pal JA, Khurshid M (2003). Chromosomal abnormalities as a cause of recurrent abortions: a hospital experience. J Pak Med.

[3] Carp H, Guetta S, Dorf H, Soriano D, Barkai G, Schiff E (2006). Embryonic karyotype in recurrent miscarriage with parental karyotypic aberrations. Fertil Steril.

[4] Byrne JL, Ward K (1994). Genetic factors in recurrent abortion. Clin Obstet Gynecol.

[5] Gardner RJ, Sutherland GR (1996). Chromosome abnormalities and genetic counseling.

[6] Summers AM, Huang T, Meier C, Wyatt PR (2003). The implications of a false positive second- trimester serum screen for Down syndrome. Obstet Gynecol.

[7] Leung WC, Lau ETK, Lau WL, Tang R, Wong SF, Lau TK (2008). Rapid aneuploidy testing (knowing less) versus traditional karyotyping (knowing more) for advanced material age: What would be missed, who should decide?. HKMJ.

[8] (2010). Fetal and Perinatal Pathology: Report of a Joint Working Party. http://www.rcpath.org/index.%20asp.

[9] Guan XY, Trent JM, Meltzer PS (2003). Generation of band-specific painting probes from a single microdissected chromosome. Hum Mol Genet.

[10] Abuelo DN, Barsel-Bawers G, Richardson A (1988). Insertional Translocations: Report of Two New Families and Review of the Literature. Am J Med Genet.

[11] Zhang H, Xia J, Li L (1990). The high resolution G band of human chromosomes at 1200 band stage. I Chuan Hsueh Pao.

[12] Li LY, Xia JH, Dai HP (2005). Chromosome analysis of 2319 cases in genetic counseling clinic. Chin Med J (ENG).

[13] ISCN (1995). Recommendations of the international standing committee on human cytogenetic nomenclature.

[14] Warburton D, Fraser FC (1964). Spontaneous abortion risks in man: Data from reproductive histories collected in a medical genetics unit. Am J Hum Genet.

[15] Coulam CB, Clark DA, Beer AE, Kutteh WH, Silver R, Kwak J (1997). Current clinical options for diagnosis and treatement of recurrent spontaneous abortion. Clinical guidelines recommendation committee for diagnosis and treatment of recurrent spontaneous abortion. Am J Reprod Immunol.

[16] Campana M, Serra A, Neri G (2006). Role of chromosome aberrations in recurrent abortion: A study of 269 balanced translocations. Am J Medl Genet.

[17] Ashaat Neveen A (2008). Detection Chromosomal Alterations of Primary Recurrent Pregnancy Loss Using Cytogenetics Analysis. Journal of Egyptian German Society of Zoology.

[18] Asamoah A, Nandi KN, Prouty L (1998). A case of insertional translocation involving chromosomes 2 and 4. Clinical Genetics.

[19] Bond PA, Maher EJ, Lindenbaum RH (2005). Maternal 3; 13 chromosome insertion with severe pre-eclampsia. Clinical Genetics.

[20] Bettio D, Venci A, Levi Setti P (2008). Chromosomal Abnormalities in Miscarriages after Different Assisted Reproduction Procedures. Placenta.

[21] Xia J, Yin Z, Dai H, Xue Z, Chen Y, Pan Q (2000). Molecular cytogenetics study in a case with unbalanced chromosome translocation. Zhonghua Yi Xue Yi Chuan Xue Za Zhi.

[22] Al-Hassan S, Hellani A, Al-Shahrani A, Deery M, Jaroudi K, Coskun S (2005). Sperm chromosomal abnormalities in patients with unexplained recurrent abortions. Arch Androl.

[23] Vorsanova SG, Kolotii AD, Iourov IY, Monakhov VV, Kirillova EA, Soloviev IV (2005). Evidence for high frequency of chromosomal mosaicism in spontaneous abortions revealed by interphase FISH analysis. J Histochem Cytochem.

[24] Appelman Z, Furman B (2005). Invasive genetic diagnosis in multiple pregnancies. Obstet Gynecol Clin North Am.

